# Examining the longevity of trade partnerships: A duration analysis of Ethiopia’s international trade flows

**DOI:** 10.1371/journal.pone.0334363

**Published:** 2026-03-13

**Authors:** Abiyot G. Gebre, Amsalu Dachito Chigeto

**Affiliations:** 1 Faculty of Business, Economics and Social Sciences, University of Kiel, Kiel, Germany; 2 Department of Economic, Jimma University, Jimma, Ethiopia; University of Salamanca, SPAIN

## Abstract

This study examines the longevity of Ethiopia’s trade partnerships duration and its determinants using a survival analysis. While the existing literature extensively studies the determinants of trade volume, this study uniquely investigates how long bilateral trade ties persist and what determines their duration, with particular emphasis on Ethiopia. Employing bilateral export data from 1997 to 2023, combined with gravity-based variables and rich institutional and macroeconomic indicators, the analysis applies random effect parametric survival estimates to capture the timing and risk of trade relationship dissolution. We find that initial export volumes and institutional quality significantly enhance trade longevity, Geographic proximity exhibits significant effects: shared borders are associated with longer trade durations, whereas longer distance shortens the duration. While Ethiopia’s own-economic size correlates with shorter trade survival, larger partner economies tend to sustain longer trade relationships. Moreover, regional dynamics reveal that Sub-Saharan African partners demonstrate greater persistence in trade links. The findings carry important policy implications for fostering sustainable trade growth and economic resilience in Ethiopia and comparable economies, underscoring the importance of strengthening institutions, upgrading infrastructure, and cultivating cooperative regional arrangements to reinforce durable trade partnerships.

## 1. Introduction

International trade has long played a critical role in shaping economic development and stability for nations worldwide. As globalization progresses, the importance of durable trade relationships becomes increasingly evident, underpinning economic resilience, market stability, and long-term growth [1,2]. Trade partnerships, particularly those characterized by sustained interactions over extended periods, foster trust, facilitate market integration, and promote economies of scale, thereby accelerating development prospects [3–5]. Conversely, short-lived or unstable trade ties can hinder economic progress by introducing uncertainty and exposing economies to volatile external shocks [6]. Moreover, when trade relationships are fragile or frequently disrupted, firms and countries face higher risks and costs in planning investments, production, and market strategies [7]. This uncertainty reduces incentives for specialization, innovation, and efficiency gains that come from stable, long-term trade partnerships. Furthermore, unstable trade ties can make economies more vulnerable to sudden external shocks, such as global demand fluctuations, political changes, or regulatory shocks, which can abruptly halt trade flows and disrupt supply chains.

Despite the broad recognition of the importance of trade relationship durability, scholarly understanding of their temporal dynamics remains underdeveloped. Existing research predominantly focuses on determinants of trade volumes and the formation of initial linkages, often neglecting how long these partnerships endure and the factors driving their persistence. Nonetheless, it is worthwhile to mention the contributions of various scholars who employ duration analysis to measure the survival time of trade relationships and to identify determinants of their continuation or discontinuation [1,7,8].

Global trade analytics demonstrate the vast scale and heterogeneity of international exchanges, with world merchandise exports reaching approximately $19 trillion in 2023 [9]. Major trading economies such as China, the United States, and the European Union have cultivated extensive, often decades-long trade networks, reinforced by formal trade agreements, infrastructural investments, and diplomatic ties all of which contribute to the longevity of trading relationships. At the regional level, trade survival is challenged by geographic distance, economic complementarity, and political alignment [10]. For example, intra-African trade has traditionally lagged behind other regions due to infrastructural deficits and challenging trade policy environments [11]. Moreover, trade longevity studies have documented that shorter geographic distances between trading partners are correlated with longer-lasting trade relations [12].

Theoretical underpinnings of trade duration largely stem from gravity models, where bilateral trade flows are positively related to economic size and negatively to geographic distance [13]. However, gravity models primarily explain trade volume and do not fully capture the survival dimension of trade relationships. To address this, researchers have increasingly applied survival analysis techniques—tools originally developed in medicine and engineering—to investigate trade persistence and rupture.

Empirical evidence from duration studies highlights the significant role of trade costs, institutional quality, political stability, and economic similarity in determining trade relationship longevity. Robust institutions and political stability tend to extend trade durations by mitigating transactional risks [14], while economic shocks and policy changes may abruptly sever established ties. Research examining regional agreements and economic integration illustrates that formal trade agreements and enhanced infrastructural connectivity bolster the durability of trade partnerships. Nonetheless, much of this literature is concentrated on developed economies and aggregated global patterns, leaving a significant knowledge gap concerning the longitudinal dynamics of trade ties among developing countries, especially within Africa.

This gap is particularly salient for Ethiopia, a country undergoing rapid economic transformation alongside persistent infrastructural, political, and market access challenges. As Africa’s second-most populous nation, Ethiopia’s recent growth has been driven by sectors such as agriculture, manufacturing, and exports of textiles, coffee, and leather products [15]. Despite this progress, Ethiopia remains heavily dependent on primary commodity exports, which are vulnerable to external price and demand fluctuations, underscoring the imperative for stable, long-term trade relationships. Ethiopia’s key trading partners include China, the European Union, and neighboring African countries, with China emerging as its largest partner—bilateral trade with China rose from approximately $1.2 billion in 2010 to over $9 billion in 2022 [16]. While some of these relationships have endured for over a decade, there is limited understanding of the determinants underpinning their stability or fragility, owing to a scarcity of longitudinal, country-specific research.

Understanding the factors that influence the longevity of trade relationships is vital for developing resilient trade strategies, especially for countries like Ethiopia that are navigating rapid economic change amidst structural challenges. Existing research has highlighted the importance of institutional quality, policy stability, and infrastructural connectivity in sustaining trade ties, yet empirical evidence examining how these factors play out over time remains limited—particularly for developing economies in Africa. A duration analysis approach offers a promising avenue for unveiling the dynamics of trade partnership stability and identifying the key determinants that sustain or jeopardize these relationships. For Ethiopia, such insights could prove instrumental in designing policies that extend the lifespan of trade ties, secure markets for its exports, and promote sustainable economic growth. As Ethiopia continues its trajectory of growth and integration into global markets, understanding the stability and durability of its trade partnerships will become increasingly essential—an endeavor that can shape the future of its international trade landscape.

This research advances the literature by analyzing country-pair-specific determinants of trade duration, explicitly accounting for the occurrence of multiple trade spells per partner pair. Unlike previous studies that typically treat each trading relationship as a single continuous spell, we model repeated trade episodes as independent observations, allowing for the capture of heterogeneity and more nuanced estimation of cumulative and differential effects. Our methodology integrates both parametric and nonparametric survival estimators within a panel data framework, leveraging gravity-model variables such as geographic distance, economic size, and cultural proximity, alongside country-level characteristics including institutional quality and macroeconomic conditions. Through this comprehensive approach, we provide fresh insights into the factors shaping the persistence and disruption of trade ties, with implications for strengthening Ethiopia’s integration within global and regional trade systems.

## 2. Literature review

International trade is fundamental to economic growth and development [17,18]. The strength and stability of trade relationships influence a country’s access to markets, technology, and investment opportunities. The concept of trade partnership longevity refers to the duration over which two or more countries maintain a continuous trading relationship, from its inception to either its termination or sustained renewal [12,19]. Long-standing trade ties often imply mutual trust, institutional support, and economic interdependence, fostering stability that can withstand external shocks. Conversely, fragile or short-term relationships are more vulnerable to disruptions, which can lead to economic instability for involved nations.

Various authors have explored how international trade relationships form and evolve, primarily through the framework of a search model [20]. They propose that buyers in developed countries select suppliers in less developed countries through a process involving search, investment, and potential rematching. The model predicts that longer trade relationships are associated with higher initial transaction values, while buyers tend to start with small, low-value orders when search costs are high or supplier reliability is uncertain. Empirical evidence such as Besedeš [7] supports these predictions, showing that relationships involving homogeneous goods tend to be shorter and more prone to early termination, and that short-duration trade links are often low-valued. Similar to this argument, importers begin with small purchases due to uncertainty, increasing only if suppliers perform reliably, consistent with the traditional product life cycle [1,2]. It highlights that initial trade activities are often cautious and modest, especially when dealing with uncertain suppliers or homogeneous products, and that relationship duration is positively related to initial trade size and reliability.

Trade partnership stability is central to understanding global economic integration. For example, some trading relationships last decades, such as Japan and the United States, which have maintained high levels of cooperation since the 1950s [21]. In contrast, others are transient, often influenced by political upheaval, policy change, or economic shocks. The duration of these relationships can be affected by various factors, including economic size, institutional quality, geographic proximity, policy frameworks, and infrastructural connectivity [22,23]. The understanding of trade relationship duration has evolved from foundational economic theories. The earliest framework, the gravity model of trade [13], posits that trade volume heavily depends on the economic size of the trading partners and the distance between them. Although effective for estimating trade flow magnitude, it does not directly address the issue of the relationship’s lifespan or stability.

To analyze trade duration specifically, scholars have turned to survival analysis techniques, borrowed from fields like medicine and engineering. These models examine the “hazard rate”—the risk that a trade relationship will end at any given time—considering various covariates such as institutional quality, policy stability, and infrastructural linkages. Such models typically produce estimates like survival functions, which depict the probability that a trade relationship persists beyond a certain number of years. Another influential framework comes from transaction cost economics, which suggests that high costs of switching or establishing new trade partnerships motivate countries to maintain existing ties longer [2]. This idea emphasizes the importance of institutional and infrastructural barriers that reinforce identity and trust among trading partners. Furthermore, institutional theories highlight the formal and informal mechanisms—trade agreements, diplomatic relations, and cultural ties—that extend the lifespan of trade relationships [5].

Complementing these theories, political economy perspectives argue that external shocks, policy shifts, and geopolitical changes can shorten or lengthen trade durations. For example, periods of economic liberalization are often associated with an increase in trade relationships’ stability, whereas protectionist policies tend to destabilize existing ties. Empirical studies applying the above theoretical approaches have shed light on the key drivers of trade partnership longevity. Baier and Bergstrand [22], using a large sample of bilateral trade data, found that institutions, policy stability, and economic openness significantly increase the probability of long-lasting trade relations. They demonstrated that trade ties are more durable when countries share common institutions, such as trade agreements, or belong to regional bodies. Similarly, Besedes et al. [2] employed survival analysis on Latin American countries trade data and found that political stability, economic size, and infrastructural connectivity increase the lifespan of trade ties. Moreover, findings by Groots et al. [24] emphasized that institutional quality acts both as a facilitator of initial trade formation and a protector of existing relationships, especially in volatile economic environments. Confirming this, Nitsch [4] finds that the duration of exporting a product to Germany tends to be longer when the exporting country is economically large and geographically close, when the product has a high trade value and low elasticity of substitution, and for trade pairs that constitute a significant share of Germany’s import market and involve two-way trade.

Further examining export durations, employ non-parametric survival techniques to analyze long-term export relationships from 46 countries to the U.S., Besedeš and Prusa [1] found that developed and successful developing countries exhibit higher survival probabilities. Similarly, Nitsch [4] investigates German import durations and finds that most trade ties are short-lived, often lasting only one to three years, with duration influenced by exporter characteristics, product types, and transaction size. Despite the emphasis on product type and value, these studies largely overlook the impact of fixed costs. The wage of entry costs, such as compliance with standards and establishing distribution networks, discourages swift exit and anchors firms in foreign markets even when external conditions change. Empirical evidence from Roberts & Tybout [25] and Rauch & Watson [20] confirms that fixed entry costs significantly influence firms’ export decisions and duration in foreign markets. Their findings demonstrate that past export activity (a proxy for sunk costs) substantially increases the probability of continued participation, underscoring the role of fixed costs in shaping trade stability beyond product and buyer-supplier characteristics. These insights collectively advocate for the incorporation of fixed costs into models of trade relationship duration, providing a more comprehensive understanding of international trade dynamics [25,26].

In the African context, Wang et al [8] examined trade using a duration model and concluded that infrastructural deficits, policy inconsistencies, and political instability significantly shorten trade relationships’ durations. These factors are particularly relevant for countries with developing economies, where systemic challenges can abruptly break long-standing ties. Despite these insights, it remains unclear how these determinants interplay over time, especially under specific country circumstances, which underscores the need for country-specific, longitudinal research approaches. While extensive research has explored the determinants of trade volume and initial relationship formation, less attention has been paid to the duration aspect—particularly, the factors that foster or hinder the long-term stability of trade partnerships. The existing empirical studies tend to focus on regional trade agreements, such as NAFTA or the EU, rather than bilateral trade dynamics involving developing countries.

Another notable gap is the limited focus on empirical data for African nations, which often face unique challenges—such as infrastructural deficits, political instability, and limited institutional capacity—that compromise trade longevity [27]. Consequently, there is a lack of longitudinal studies that examine how these factors influence trade ties’ survival over extended periods. Specifically, for Ethiopia, the empirical literature remains fragmented. While some research emphasizes the country’s rapid economic growth, export expansion, and regional integration efforts, few studies analyze the stability or sustainability of these newly forming trade partnerships. Given Ethiopia’s strategic positioning in East Africa and its growing trade with China, the European Union, and neighboring countries, understanding the factors that influence the durability of these ties is crucial for policy formulation and investment decisions. Furthermore, existing models often ignore the dynamic nature of trade relationships, such as their evolution in response to changing policy landscapes or infrastructural developments. Such gaps point toward the necessity for rigorous, country-specific research employing advanced duration analysis techniques that capture both external and internal determinants of trade stability over time.

Ethiopia provides a compelling case study within this broader framework. Over the past two decades, Ethiopia has experienced notable economic growth, with GDP expanding at average rates of around 9% per annum [28]. During this period, the country transitioned from a primarily agricultural economy to one emphasizing manufacturing and export-oriented growth. Its trade has increasingly become diversified, with China emerging as Ethiopia’s top trading partner, accounting for nearly $9 billion in bilateral trade in 2022 [29]. Despite this rapid growth, the longevity of Ethiopia’s trade ties remains underexplored. On one hand, trade with China appears robust and expanding, suggesting long-term relationship stability. However, infrastructural issues—such as port congestion, transport bottlenecks, and energy shortages—pose risks to sustaining these partnerships. Political dynamics, including policy shifts, regional conflicts, and the recent economic reforms, could further influence the durability of these trade links.

Research focused on Ethiopia’s trade partnership duration could clarify how factors like infrastructural investment, institutional reforms, and geopolitical shifts influence the survival and resilience of these relationships. Such insight is vital for Ethiopia’s policymakers aiming to develop sustainable trade strategies that withstand external shocks and internal uncertainties.

## 3. Materials and methods

### 3.1. Variables and data

In this study, we leverage a comprehensive collection of secondary data sources to explore the factors influencing the duration and stability of Ethiopia’s international trade partnerships. Central to this analysis is the use of a trade gravity dataset—an extended and enriched version of the classical gravity model variables—capturing bilateral trade flows, distance metrics, economic sizes, and additional contextual factors [30,31]. The core dataset employed is the UN Comtrade Database, which compiles detailed trade flow data disaggregated by partner country, and time from 1997 to 2023, selected based on UN Comtrade data availability for bilateral trade flows at yearly frequency. Then it is complemented by macroeconomic indicators such as GDP and population figures from the World Bank’s World Development Indicators [32]. These variables serve as the foundation for the gravity model analysis, enabling us to examine how economic size, geographical proximity, trade agreements, historical ties, and institutional factors influence the longevity of Ethiopia’s trade partnerships.

Following Linder’s hypothesis [33] that countries with similar demand patterns tend to trade one another, we include country similarity index was included as a predictor.


$$SML=ln\left\{1-{\left(\frac{Y_{i}}{Y_{i}+Y_{j}}\right)}^{\text{2}}-{\left(\frac{Y_{j}}{Y_{i}+Y_{j}}\right)}^{\text{2}}\right\}$$ (1)

Where Y_i_ and Y_j_ are GDP of the exporter and destination countries respectively.

Additionally, following empirical works such as Besedeš [7], we include variables such as bilateral distance, shared borders, and colonial history, sourced from the CEPII Gravity Dataset [34], along with variables reflecting trade facilitation and institutional quality (IQ) from the World Bank’s Worldwide Governance Indicators reports. IQ is constructed from World Bank WGI six governance indicators—Voice & Accountability, Political Stability, Government Effectiveness, Regulatory Quality, Rule of Law, and Control of Corruption—aggregated via principal components analysis (PCA). The first principal component (PC1) captures 95%+ of common variance across dimensions, serving as a summary institutional quality index standardized to mean zero and unit variance. These rich, multidimensional data sources allow us to identify key determinants that prolong or shorten the duration of trade partnerships, providing insights into the factors that foster resilient trade relations. For details, refer Table 1.

**Table 1:** Data description (type and sources).

Variable	Description	Sources
Export	Initial Bilateral export volume of Ethiopia to its partner countries	COMTRADE
$Y_{i}\backslash )$	Log of Real GDP of Ethiopia	WDI
$Y_{j}\backslash )$	Log of Real GDP of destination countries	WDI
${POP}_{i}\backslash )$	Log of population of Ethiopia	WDI
${POP}_{j}\backslash )$	Log of population of destination countries	WDI
Distance	Log of geographic distance between Ethiopia and partner countries	WDI
Border	Dummy variable (= 1, if border is shared between Ethiopia and its trade partner)	CEPII
COMESA	Dummy variable (= 1, if destination country is member of COMESA)	WTO
SSA	Dummy variable (= 1, if destination country is located in SSA)	WDI
GN	Dummy variable (= 1, if destination country is located in the global north)	World population review
SIMIL	Similarity index between Ethiopia and its trade partner	Derived
${IQ}_{i}\backslash )$	Institutional quality of Ethiopia (computed using PCA)	WB
${IQ}_{j}\backslash )$	Institutional quality of destination country (computed using PCA)	WB
Inst_Dis	Institutional distance between Ethiopia and its partner (absolute difference of institutional quality of the two countries)	Derived
CC	Control of Corruption	WB
GE	Government Effectiveness	WB
PS	Political Stability and Absence of Violence/Terrorism	WB
RQ	Regulatory Quality	WB
RL	Rule of Law	WB
VA	Voice and Accountability	WB

One important aspect of trade survival models is how “trade spell” is treated, which generally refers to a continuous period during which a trade relationship or export activity between two trading partners remains active without interruption. As expected, many countries engage in “multiple trade spells,” meaning several distinct episodes or periods of trade activity between the same partners that start and stop over time. Thus, instead of one continuous, uninterrupted trade relationship, there are multiple discrete intervals or spells of trade engagement. Following Peng & Wang [35], we treat these trade spells as mutually independent to ensure the robustness of the survival estimates.

### 3.2. The model

The survival analysis framework is especially appropriate for this study because it explicitly models the *timing* of trade relationships. Unlike static models that focus solely on whether regression occurs, survival models estimate the *hazard rate*—the instantaneous probability that democracy will regress at a particular time, given that it has survived up to that point [36]. This dynamic approach aligns with the research goal of understanding *when* bilateral trade ties are most vulnerable and *what* factors influence the risk at different stages of the macroeconomic and structural changes. Prior research, such as Hess & Persson [19], have successfully employed hazard models to analyze trade duration and stability, demonstrating their suitability for capturing complex temporal dynamics in the bilateral trade ties.

Survival models can be classified into three distinct types—parametric, semi-parametric, and nonparametric—based on the underlying assumptions made about the data and the model.

- Parametric models assume a specific distributional form (e.g., exponential with constant hazard h(t)=λ, or Weibull with monotone hazard $h(t)=\lambda pt^{p-1}$). where λ is the hazard rate. They offer precise interpretation but require correct specification.
- Semi-parametric models, like the Cox proportional hazards (PH) model, specify $h(t|X)=h_{\text{0}}(texp(X\beta )$, where $h_{\text{0}}(t)$ is an unspecified baseline hazard. They avoid baseline assumptions but rely on proportional hazards and continuous-time data.
- Non-parametric models, such as Kaplan-Meier, estimate the survival function $\hat{S}(t)=\prod_{t_{i}\leq t}(1-d_{i}/n_{i})$ without functional forms, ideal for descriptive visualization but limited for covariate analysis.

We employ a parametric panel survival model given our panel-structured UN Comtrade data, which tracks repeated yearly observations on bilateral trade pairs (country-pairs as units i, durations as time periods t). This extends standard survival analysis by specifying a parametric hazard or survival function—typically exponential, Weibull, Gompertz, or accelerated failure time (AFT)—while addressing intra-unit dependence, right-censoring, time-varying covariates $X_{it}$, and unobserved heterogeneity via frailty terms $v_{i}$ or random effects $\alpha_{i}$. For trade pair i at time t, we observe failure times or censoring alongside covariates, with the hazard often specified as $h_{it}(t|X_{it},v_{i})=v_{i}h_{\text{0}}(texp(X_{it}\beta )$, where $h_{\text{0}}(t)$ follows a Weibull form for monotone risks. This approach suits discrete yearly intervals, avoiding Cox PH’s continuous-time assumption and proportional hazards violations seen in trade data [19,36], while enabling covariate analysis unavailable in Kaplan-Meier.

Empirical studies strongly validate parametric panel survival models with frailty for bilateral trade duration analysis, particularly given discrete yearly data. Hess and Persson [19] demonstrate that continuous-time Cox models produce biased estimates in trade contexts, advocating discrete-time parametric approaches with random effects for exporter-product pairs, which reveal persistent short-duration trade patterns and unobserved heterogeneity trends over 1962–2006. Similarly, [36] document proportional hazards violations in export survival, while parametric Weibull frailty extensions—handling intra-pair correlation via gamma-distributed terms—outperform in panel settings with censoring and time-varying covariates like GDP or RTAs. Recent applications, including Ethiopian manufacturing export survival [37] and developing-country flows [38], confirm these models’ robustness for identifying hazard drivers such as initial trade value and market access. Weibull-gamma frailty specifications further excel in capturing monotone hazards and clustering, as validated across survival literature.

In our setup, the hazard for trade pair i at time t follows a proportional hazards form:


$$h\left(t|X_{it,}u_{i}\right)=h_{\text{0}}\left(t|{u_{i})exp(X}_{it,}\beta \right)$$ (2)

where $h_{\text{0}}(t\;|\;\alpha_{i})$ is the baseline hazard incorporating unit-specific effect $\alpha_{i}$, and β are log-linear coefficients. Alternatively, an accelerated failure time (AFT) formulation assumes


$$\in_{it}$$ (3)

with $\in_{it}$ drawn from a specified distribution (extreme value, normal, logistic). Time-varying covariates enter $X_{it}$; time-invariant ones interact with $\alpha_{i}$. Unobserved heterogeneity—key in panel trade data—is addressed via multiplicative frailty in the PH framework:


$$\in_{it}$$ (4)

where $v_{i}\sim \Gamma (\theta ,\theta )$ (mean 1, variance 1/θ). This induces intra-pair correlation, with estimation via maximum likelihood and Gauss-Hermite quadrature integrating out frailties. We select Weibull baseline $h_{\text{0}}(t)=\lambda pt^{p-1}$ for its monotone hazards matching trade durability patterns (early failures from shocks, later stabilization), outperforming exponential per AIC.

Identification in parametric panel survival models hinges on the interplay between covariates, baseline specification, and unobserved effects. If time-invariant regressors are collinear with unit effects, their coefficients may be unidentifiable under certain specifications; this motivates either using random effects with distributional assumptions or adopting conditional (within-unit) likelihoods that purge fixed effects. Estimation methods include maximum likelihood with numerical integration (e.g., Gauss-Heather or Gauss-Hermite quadrature for random effects), marginal likelihoods with Laplace approximations, or Bayesian approaches with data augmentation. Interpreting parameters follows the chosen form: in PH models, exp(β) represents hazard ratio effects of covariates; in AFT models, effects are multiplicative on the event time scale (e.g., percent change in expected survival time). Model selection and validation typically involve information criteria adapted to survival contexts (e.g., AIC, BIC), likelihood ratio tests, and out-of-sample predictive performance, with attention to censoring mechanisms and potential informative censoring.

### 3.3. Estimation and visualization strategies

We begin with Kaplan-Meier (KM) survival estimates and Nelson-Aalen (NA) hazard plots, which provide non-parametric descriptives of raw bilateral trade duration patterns from UN Comtrade. These empirical curves facilitate visual assessment of censoring distribution, baseline hazard shape, and group differences (e.g., by RTA status, and other shared attributes), while validating key assumptions—such as monotone decreasing survival consistent with Weibull specification—prior to parametric estimation. We then estimate the parametric panel survival model using random effects with frailty, well-suited for time-to-event data featuring clustering at the country-pair level. This approach specifies a Weibull baseline hazard $h_{\text{0}}(t)=\lambda pt^{p-1}$ and incorporates gamma-distributed frailty terms to capture unobserved heterogeneity, effectively handling right-censoring and intra-pair correlation across yearly observations [19]. It delivers robust covariate effects even when some trade relationships remain active at study end

## 4. Results and discussions

This study analyzes the longevity of Ethiopia’s bilateral trade using a two-step approach. The data analysis begins with descriptive exploration to characterize the dataset and uncover initial patterns, followed by formal modeling to quantify the determinants of trade survival. Descriptive statistics: We compute summary statistics (means, medians, ranges, and dispersion) to describe key variables and their distributions. Survival-analysis visuals: We present survival-model–based graphs and tables (e.g., Kaplan–Meier–type trajectories and related confidence bands) to illustrate the duration and stability of trade ties across different partner groups. These descriptive tools provide an intuitive, data-driven overview of the longevity patterns and help identify potential heterogeneity across subsamples. We move beyond description to quantify how bilateral characteristics influence trade survival. The core specification uses random-effects parametric survival models to account for unobserved heterogeneity across destinations and origin-year effects. This step yields estimates of how bilateral attributes (e.g., institutional distance, economic size, distance, regional indicators) affect the hazard or duration of trade ties, while controlling for clustering and potential endogeneity through random effects.

Table 2 reveal substantial dispersion in trade outcomes and relational characteristics. Trade spell exhibits a wide range (mean 5.58, SD 7.13; min 1, max 27), signaling considerable variability in the duration or persistence of trade relationships across observations. Export values are highly skewed and variable. While institutional quality indicators (IQ_i mean 0.173; IQ_j = 0) and a broad spectrum of institutional distance (Inst_Dis mean 2.077) imply notable heterogeneity in governance and risk environments.

**Table 2:** Descriptive Statistics.

Variable	Obs	Mean	Std. Dev.	Min	Max
Trade spell (in year)	556	5.5827	7.1308	1	27
Export	556	1360861.8	6895201	501.668	94000000
$Y_{i}\backslash )$	556	24.225	.604	23.468	25.448
$Y_{j}\backslash )$	539	23.866	1.881	17.872	30.119
${POP}_{i}\backslash )$	556	18.253	.207	17.933	18.673
${POP}_{j}\backslash )$	556	15.425	1.921	9.223	20.93
Distance	556	6759.978	3877.038	559	16774
Border	556	.018	.133	0	1
COMESA	556	.068	.253	0	1
SSA	556	.255	.436	0	1
GN	556	.095	.294	0	1
SIMIL	539	−1.514	.937	−5.926	−.693
${IQ}_{i}\backslash )$	556	.173	1.417	−4.247	1.869
${IQ}_{j}\backslash )$	556	0	2.241	−5.16	6.042
Inst_Dis	556	2.077	1.662	.008	9.835
CC	556	−.203	.855	−1.75	2.435
GE	556	−.21	.839	−2.221	2.408
PS	556	−.121	.878	−3.18	1.589
RQ	556	−.191	.848	−2.383	1.985
RL	556	−.264	.847	−2.301	1.995
VA	556	−.168	.863	−2.238	1.668

**Fig 1:**
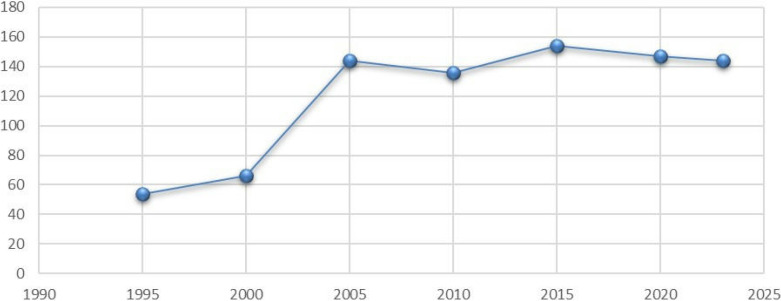
No. of Export Destinations by year.

Fig 1 illustrates the trend in the number of export destinations for Ethiopia from 1995 to 2020. There is a notable increase in the number of export destinations during this period, with a sharp rise observed between 2000 and 2005, indicating a period of expansion in Ethiopia’s export markets. Although there are fluctuations in the number of destinations after 2005, the overall trend remains relatively stable, hovering around 140–150 destinations. This suggests that Ethiopia has maintained a diverse export market, which is beneficial for economic stability and growth by reducing dependence on a limited number of markets. The slight decline towards 2020 could imply a temporary consolidation or challenges in opening new markets, but the overall pattern highlights steady growth in Ethiopia’s export diversification efforts over the years.

**Fig 2:**
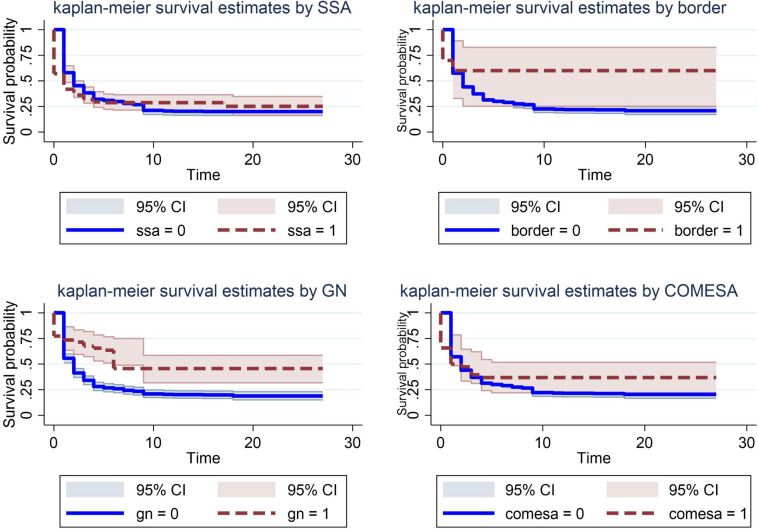
Kaplan-Meier survival estimates across different groups.

The series of Kaplan-Meier survival plots in Fig 2 illustrate the longevity of Ethiopia’s bilateral trade with different partner groups. Across all panels, the survival probability starts near one and declines quickly in the early periods, indicating that a large share of trade ties terminates soon after they begin. This rapid early drop suggests that many Ethiopia–partner relationships are short-lived, while a smaller subset persists over longer horizons. Several patterns stand out. First, bilateral ties with SSA partners show a steeper initial decline but then level off sooner, implying a relatively higher rate of early-term terminations but fewer very long-lived relationships within that bloc. In contrast, trade ties with COMESA and GN destinations exhibit somewhat different trajectories: COMESA displays higher survival probabilities over the mid-to-long term, suggesting more durable ties within that regional bloc. The GN panel, by comparison, indicates more pronounced fragility, with a faster erosion of survival probability, pointing to greater challenges in sustaining long-run trade with Global North partners.

**Fig 3:**
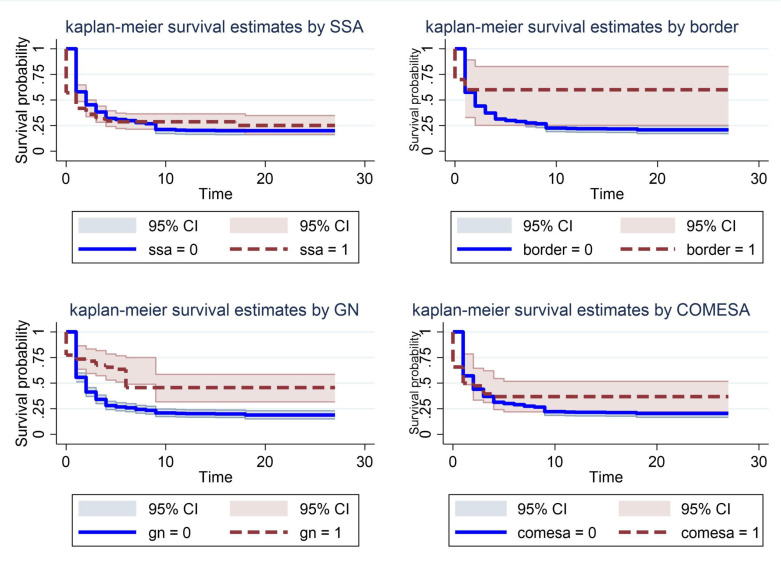
Kaplan-Meier-like survival (observed vs predicted plots by subgroups).

Fig 3 shows a close alignment between observed and predicted survival across four subgroups (SSA, GN, border, and COMESA) over 27-time units, with survival probabilities starting high and declining over time; in most panels, the predicted curves track the observed trajectories fairly well, indicating good model fit.

**Fig 4:**
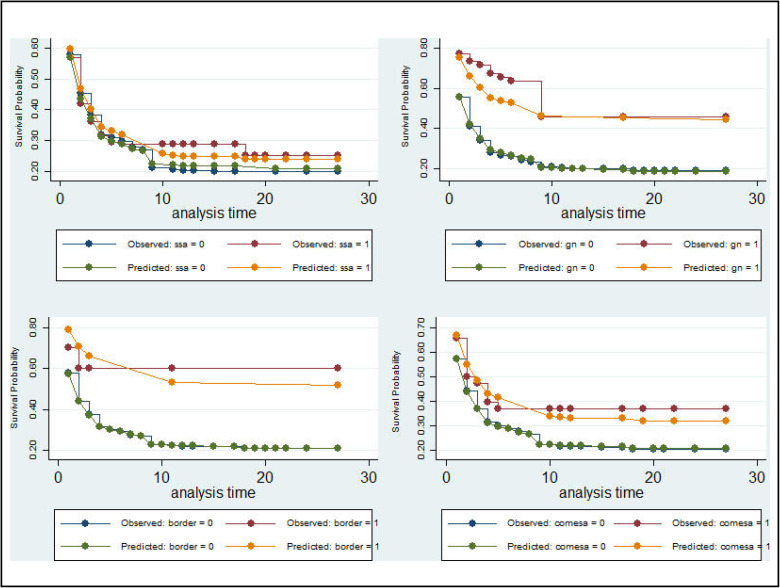
Kaplan–Meier like survival trajectory.

Fig 4 plots a Kaplan–Meier like survival trajectory derived from the L-table, tracking Ethiopia’s bilateral trade longevity over time (t). The survival probability starts near one and declines gradually in early periods, indicating most trade ties endure initially. A pronounced drop occurs around t ≈ 16–18, revealing a cluster of ties that terminate in the mid horizon, followed by a plateau at a lower level. The tail eventually approaches zero, suggesting few long-lasting bilateral relationships persist beyond the later horizon. Overall, the graph indicates that while many trade links are short-lived, a subset remains durable for longer periods before ultimately ending.

This Nelson–Aalen table, (Table 3), shows the estimated survival trajectory with cumulative events and the corresponding confidence bounds. Across early times, the survival probability declines from about 0.58, with relatively wide standard errors (around 0.045), reflecting sampling variability. The hazard-like increments decrease as time progresses, with notable sharp drops around time points where many events occur (e.g., at times 9–11 and 27). By the later observations, few events occur and the estimated survival stabilizes toward the 0.15–0.31 range, though the tail has fewer subjects and larger uncertainty. Overall, the data indicate a rapid initial decline in survival followed by a slower decay and increasing uncertainty as the number at risk diminishes.

**Table 3:** Nelson–Aalen (hazard-like) estimates.

Time	Beg. Total	Fail	Net Lost	Survival Function	Std. Error	[95% Conf. Int.]
1	119	50	0	0.5798	0.0452	0.4860 0.6626
2	69	8	0	0.5126	0.0458	0.4196 0.5980
3	61	6	0	0.4622	0.0457	0.3708 0.5486
4	55	3	0	0.4370	0.0455	0.3468 0.5236
5	52	2	0	0.4202	0.0452	0.3309 0.5067
6	50	1	0	0.4118	0.0451	0.3229 0.4983
7	49	2	0	0.3950	0.0448	0.3072 0.4813
8	47	1	0	0.3866	0.0446	0.2994 0.4728
9	46	18	0	0.2353	0.0389	0.1637 0.3145
11	28	0	1	0.2353	0.0389	0.1637 0.3145
17	27	0	1	0.2353	0.0389	0.1637 0.3145
18	26	1	0	0.2262	0.0384	0.1558 0.3049
19	25	0	1	0.2262	0.0384	0.1558 0.3049
24	24	0	2	0.2262	0.0384	0.1558 0.3049
27	22	0	22	0.2262	0.0384	0.1558 0.3049

### 4.1. Regression result

Random-effects parametric survival models are well-suited for bilateral trade longevity data (Ethiopia and partner countries, 1997–2023) because they accommodate time-to-event outcomes (duration of trade ties) while accounting for unobserved heterogeneity across dyads and over time. The shared frailty component captures unmeasured factors common to each country pair, such as institutional ties or bilateral history, which can induce correlations within dyads and violate independence assumptions. Our parametric forms (exponential, lognormal, gamma) offer flexibility to model the underlying hazard or failure-time distribution—allowing the hazard to be constant, increasing then decreasing, or otherwise shaped as trade durations evolve—while still yielding interpretable effects of covariates (e.g., distance, policy barriers, GDP, border status) on the duration of trade relationships. This approach provides efficient estimates with fully specified likelihoods, enables hypothesis testing via Wald/LR statistics, and supports out-of-sample prediction and counterfactual scenarios critical for understanding the persistence and dissolution of trade ties in a dynamic, panel-like bilateral context.

The Wald tests across all four distributional specifications (exponential, loglogistic, lognormal, gamma) are significant (Wald chi-square with p < 0.01 in each case). This indicates that, under every assumed distribution, the covariates collectively have substantial explanatory power, and the null hypothesis that all 14 restrictions are zero is strongly rejected. In other words, the model covariates jointly matter for explaining the outcome under each distributional form. In contrast, the Likelihood Ratio (LR) tests show a more consistent pattern. For the exponential and gamma models, the LR tests are significant (e.g., χ2(2) with *p* < f 0.01 for gamma and p < 0.05 for exponential), suggesting that the current model provides a meaningful improvement over the nested baseline model. However, for the loglogistic and lognormal specifications, the LR tests are not significant (χ2(2) around 2–3 with p > 0.01), implying weaker evidence that the current specification markedly improves the fit over the restricted baseline in the LR sense. Thus, our subsequent discussions refer to the gamma specifications (column 4 Table 4).

**Table 4:** Random effect parametric survival estimates (destinations = all).

	(1)	(2)	(3)	(4)
VARIABLES	Exponential	Loglogistic	Lognormal	Gamma
Export	−1.72e-07***	1.56e-07***	1.06e-07***	1.37e-07***
	(5.13e-08)	(4.31e-08)	(2.71e-08)	(4.17e-08)
$Y_{i}\backslash )$	2.537***	−1.931***	−1.918***	−2.405***
	(0.615)	(0.521)	(0.517)	(0.520)
$Y_{j}\backslash )$	−0.344***	0.267***	0.274***	0.360***
	(0.0827)	(0.0752)	(0.0732)	(0.0823)
${POP}_{i}\backslash )$	−6.232***	4.781***	4.710***	6.198***
	(1.853)	(1.560)	(1.541)	(1.574)
${POP}_{j}\backslash )$	−0.0899	0.0899	0.0964	0.0722
	(0.0749)	(0.0658)	(0.0647)	(0.0739)
Distance	9.73e-05***	−8.75e-05***	−8.92e-05***	−9.63e-05***
	(1.91e-05)	(1.69e-05)	(1.68e-05)	(1.95e-05)
Border	−1.234*	1.089*	1.018**	1.279**
	(0.666)	(0.572)	(0.516)	(0.607)
COMESA	−0.523*	0.273	0.310	0.493*
	(0.285)	(0.255)	(0.234)	(0.280)
SSA	−0.583***	0.226	0.305*	0.579***
	(0.208)	(0.189)	(0.182)	(0.211)
GN	0.115	−0.0704	−0.0694	−0.0792
	(0.304)	(0.264)	(0.255)	(0.298)
SIMIL	0.297***	−0.229***	−0.234***	−0.277***
	(0.0794)	(0.0689)	(0.0665)	(0.0774)
${IQ}_{i}\backslash )$	−0.455***	0.378***	0.389***	0.443***
	(0.0483)	(0.0471)	(0.0458)	(0.0424)
${IQ}_{j}\backslash )$	−0.120***	0.102***	0.0999***	0.107***
	(0.0409)	(0.0355)	(0.0344)	(0.0385)
Inst_Dis	−0.0688*	0.0335	0.0289	0.0744**
	(0.0369)	(0.0352)	(0.0345)	(0.0329)
Constant	60.46***	−47.13***	−46.37***	−63.19***
	(19.89)	(16.81)	(16.61)	(17.05)
Wald chi2(14)	322.74***	255.52***	273.91***	358.08***
LR (chi2(2))	9.17**	2.69	2.09	27.00***
N	539	539	539	539
No. id	186	186	186	186

Standard errors in parentheses; *** p < 0.01, ** p < 0.05, * p < 0.1

***Note****: The exponential model (column 1) reports on the hazard (or hazard ratio) scale — i.e., a Cox proportional hazards framework. In this specification, the coefficient is interpreted as the instantaneous risk of the event at any time point changing by a factor* $HR=e^{\beta }$ *per unit change in the covariate, assuming proportional hazards. By contrast, the log-logistic, lognormal, and gamma models (i.e., the accelerated failure time, or AFT, family) report on the survival-time scale. In these models, coefficients are interpreted as effects on the log of survival time (i.e., the log of time until the event), which can be expressed as time ratios (TR) with* $TR=e^{\beta }$ *for a one-unit change in the covariate.*

The apparent opposite signs between the exponential regression and the other three models arise from their distinct time scale interpretations. The exponential model assumes a constant hazard h0(t) = λ, so the linear predictor enters the log-hazard: a positive coefficient increases the hazard and shortens survival, while a negative one reduces the hazard. By contrast, the other three model (being the family of accelerated-failure-time (AFT), specifications) yields a time-varying, non-monotone hazard where the effect of covariates on μ translates into longer or shorter expected time to event in a nonlinear way; a positive coefficient raises μ, often implying longer survival in terms of time-to-event, but the corresponding impact on hazard depends on the entire time trajectory. Thus, the sign of a coefficient in the lognormal model does not simply map to the hazard direction as it does in the exponential model, leading to what can appear as opposite signs across model specifications.

Referring to column (4) of Table 4, the coefficient on initial export volume is positive (β = 1.37e-07, SE = 4.17e-08) and statistically significant. This implies that larger initial export flows from Ethiopia to destination countries are associated with longer survival of trade ties—in other words, stronger early trade intensity corresponds to more durable trade relationships over time. From a practical standpoint, high initial export levels may signal deeper economic complementarities, integrated supply chains, and firmer partner commitments. Such factors can reduce the likelihood of exit from the trade relationship and extend the duration of bilateral ties. This result aligns with the broader literature, including Besedeš [7], which finds that larger initial import volumes are associated with longer durations. Taken together, large initial trade volumes likely reflect a combination of established trading routines, credible sunk costs in the form of shared logistics and standards, and stronger mutual incentives to maintain ongoing trade.

The coefficient on $Y_{i}$ is negative and significant (−2.405, SE = 0.520), indicating that higher GDP in Ethiopia ($Y_{i}$) is associated with shorter survival times of bilateral trade, all else equal. In contrast, $Y_{j}$ is positive and significant (0.360, SE = 0.0823), suggesting that higher GDP in the destination country ($Y_{j}$) is associated with longer trade durations (i.e., a lower hazard of dissolution) within this time-to-event framework. The negative coefficient for Ethiopia’s GDP suggests that, for Ethiopian firms, a larger domestic market may enable quicker establishment or maintenance of trade links to capture higher volumes, shortening the time to maturation. By contrast, the positive coefficient for destination-country GDP indicates that larger destination markets tend to consolidate and sustain trade ties more quickly, reducing the observed duration from initiation to stable trade. Population indicators display a stark asymmetry: ${POP}_{i}$ is strongly positive (*β*=6.198, SE = 1.574), pointing to longer trade durations when Ethiopia’s population is larger, while ${POP}_{j}$ is small and statistically indistinct.

Moreover, distance *(geographic distance between Ethiopia and its partner)* emerges as a robust and economically meaningful determinant in our model. *Distance* coefficient is negative and significant (−9.63e − 05, SE ≈ 1.95e − 05). This indicates that greater geographic distance between Ethiopia and a destination country is associated with shorter trade durations (i.e., a higher hazard of dissolution). The magnitude, while small on a per-kilometer basis, accumulates meaningfully across large distance gaps, consistent with the idea that longer distances raise transport and transaction costs, dampening the persistence of trade ties over time. This pattern aligns with classic gravity intuition: distance inhibits the longevity of bilateral trade relationships when viewed through a time-to-event lens. This finding aligns with Obashi [12] who documented that hazard rates rise as geographic distance grows.

Shared border shows a positive and statistically meaningful association with longer survival times in this model (*β* ≈ + 1.279, SE ≈ 0.607; p < 0.05). This suggests that sharing a border with Ethiopia is linked to longer-lasting trade ties, or a lower hazard of dissolution, after accounting for other factors. Proximity via direct land connection likely facilitates lower transport costs, easier negotiation, and stronger logistical ties, contributing to more durable bilateral trade relationships within the time-to-event framework.

COMESA, SSA, and GN identify whether the trade destination belongs to the COMESA bloc, the SSA region, or the Global North (GN), respectively. The coefficients for these destination-type indicators illuminate how geographical and regional groupings influence the persistence of trade. The COMESA coefficient is not statistically significant at conventional levels, indicating that being COMESA membership plays minimal role to trade survival. This finding contrasts with Peng & Wang [35], who report higher trade survival among partners of China when the partners share a trade bloc. In contrast, the SSA indicator is significant, implying that trade with Sub-Saharan African destinations yields substantially higher trade survival relative to the baseline result. This likely reflects the advantages of proximity, regional integration, or shared macroeconomic cycles that stabilize trade relationships. The GN coefficient is −0.0792 with a standard error of 0.298 and is not statistically significant, suggesting no robust difference in trade survival for Global North destinations. The finding is contrary to the long-held trade relations that the Global Norths have with LDCs and contrasts with Rauch & Watson [20], who highlight that GNs prefer to stick with the existing trade ties with the Global South rather than seeking new partners from the South due to concerns about uncertainties.

In addition to the regional destination effects, the model estimates for SIMIL, IQ, and INST_DIS illuminate how bilateral similarity and institutional characteristics shape Ethiopia’s trade survival. The SIMIL coefficient is negative and significant (−0.277, SE = 0.0774), indicating that greater similarity between Ethiopia and its partner country is associated with shorter-lasting or less persistent trade ties, holding all else constant. Mechanically, higher similarity in this specification may reflect overlapping production capabilities and product portfolios that reduce the distinct advantages needed to sustain durable trade relationships.

Institutional quality of exporter and destination, respectively are both positive and significant across each model, with around 0.389–0.443 and around 0.099–0.107 (all significant at conventional levels). This pattern suggests that higher institutional quality on both sides of the trade pair enhances trade survival. For exporters, better domestic institutions can reduce risks, improve the reliability of contract enforcement, and facilitate smoother logistics. For the destination, stronger institutions can lower governance friction, improve contract compliance, and provide more predictable trading conditions. The joint positive effect implies that complementary institutional strength—clear rules, credible policy, property rights protection, and efficient bureaucratic processes—supports more durable trade links between Ethiopia and its partners than the individual effects.

Similarly, INST_DIS (institutional distance between exporter and destination – the institutional quality difference in absolute terms) is positive and statistically significant (≈ 0.074 with SE ≈ 0.033) indicating a greater institutional distance between Ethiopia and its trade partners is associated with higher trade survival, which, at first glance, may seem counterintuitive. A plausible interpretation is that INST_DIS could capture asymmetries or complementarities rather than misalignment: pairs with meaningful differences in institutional quality might engage in more carefully designed, risk-managed trade arrangements, or specialized export flows may persist precisely because institutions complement in a way that reduces perceived risks for ongoing trade. Moreover, the findings go in line with that of [23]. The positive and significant effect warrants a more robust reading and suggests that institutional distance is not simply a barrier but can be a context-dependent determinant of trade durability.

### 4.2. Robustness check

To assess the robustness of our estimation framework, we perform a two-tier subsample analysis focused on longevity across destinations. In Table 5, we estimate the sub-sample model using Global South destinations only, evaluating whether the estimated longevity patterns hold within this focused group. We then apply the same modeling approach to these subgroups to examine consistency of the estimated parameters. In Table 6, we extend the robustness check by estimating alternative sub-samples using random-effects parametric survival models with destinations treated as the all destinations. Specifically, columns (1)–(3) report the estimated coefficients for the 2000–2024 sub-sample, and columns (4)–(6) report the corresponding estimates for the 1997–2019 sub-sample. A further dimension of robustness is introduced by assessing whether the COVID years (approximately 2020–2021, and surrounding periods) alter the original estimates; this is done by comparing pre-COVID and whole sample (COVID-period included), estimates within the same framework to detect any shifts in longevity effects. This strategy tests the stability of our key findings to sub-sample choice, pandemic shocks, and model specification, thereby supporting the robustness of our conclusions.

**Table 5:** Random effect parametric survival estimates (destinations = Global south).

	(1)	(2)	(3)	(4)
VARIABLES	Exponential	Loglogistic	Lognormal	Gamma
Export	−2.90e-07***	3.13e-07***	2.39e-07***	2.43e-07***
	(7.96e-08)	(6.44e-08)	(4.56e-08)	(6.42e-08)
$Y_{i}\backslash )$	3.212***	−2.197***	−2.338***	−2.980***
	(0.650)	(0.549)	(0.543)	(0.539)
$Y_{j}\backslash )$	−0.245***	0.192***	0.172***	0.264***
	(0.0781)	(0.0607)	(0.0571)	(0.0764)
${POP}_{i}\backslash )$	−8.595***	5.719***	6.058***	8.235***
	(1.968)	(1.652)	(1.610)	(1.638)
${POP}_{j}\backslash )$	−0.152**	0.102*	0.131**	0.131*
	(0.0726)	(0.0576)	(0.0560)	(0.0712)
Distance	0.000127***	−9.84e-05***	−0.000101***	−0.000124***
	(1.87e-05)	(1.43e-05)	(1.42e-05)	(1.89e-05)
Border	−1.433**	0.905	0.890*	1.468**
	(0.674)	(0.573)	(0.469)	(0.608)
COMESA	−0.734***	0.337	0.395**	0.713***
	(0.275)	(0.220)	(0.198)	(0.267)
SIMIL	0.316***	−0.184***	−0.186***	−0.296***
	(0.0840)	(0.0655)	(0.0623)	(0.0811)
${IQ}_{i}\backslash )$	−0.449***	0.348***	0.362***	0.442***
	(0.0494)	(0.0479)	(0.0462)	(0.0424)
${IQ}_{j}\backslash )$	−0.119**	0.111***	0.117***	0.102**
	(0.0461)	(0.0365)	(0.0353)	(0.0439)
Inst_Dis	−0.0472	0.0237	0.0257	0.0459
	(0.0417)	(0.0353)	(0.0352)	(0.0365)
Constant	85.56***	−56.11***	−58.76***	−84.77***
	(21.16)	(17.71)	(17.01)	(17.74)
Wald chi2(12)	276.66***	215.67***	244.10***	325.11***
LR (chi2(2))	11.13**	0.06	1.8e-12	31.13***
N	486	486	486	486
No. id	161	161	161	161

Standard errors in parentheses; *** p < 0.01, ** p < 0.05, * p < 0.1.

**Table 6:** Sub-sample random effect parametric survival estimates (destinations = All).

	2000 - 2024			1997 - 2019		
	(1)	(2)	(3)	(4)	(5)	(6)
VARIABLES	exponential	loglogistic	lognormal	exponential	loglogistic	lognormal
expo	−1.66e-07***	1.50e-07***	1.05e-07***	−1.77e-07**	1.72e-07***	1.02e-07***
	(5.07e-08)	(4.28e-08)	(2.73e-08)	(8.11e-08)	(6.26e-08)	(3.91e-08)
yi	2.399***	−1.849***	−1.838***	1.137	−1.198	−1.034
	(0.618)	(0.525)	(0.522)	(1.165)	(0.981)	(0.986)
yj	−0.347***	0.279***	0.285***	−0.312***	0.223***	0.229***
	(0.0832)	(0.0773)	(0.0750)	(0.0915)	(0.0762)	(0.0752)
popi	−5.639***	4.520***	4.451***	−1.296	1.998	1.471
	(1.874)	(1.583)	(1.559)	(3.822)	(3.192)	(3.199)
popj	−0.0890	0.0874	0.0934	−0.162*	0.162**	0.170**
	(0.0755)	(0.0672)	(0.0661)	(0.0859)	(0.0706)	(0.0694)
distance	9.36e-05***	−8.69e-05***	−8.86e-05***	9.65e-05***	−9.18e-05***	−9.10e-05***
	(1.94e-05)	(1.76e-05)	(1.75e-05)	(1.97e-05)	(1.67e-05)	(1.67e-05)
border	−1.215*	1.119*	1.039**	−0.311	0.531	0.510
	(0.667)	(0.580)	(0.527)	(0.813)	(0.650)	(0.660)
comesa	−0.533*	0.271	0.311	−0.574*	0.304	0.346
	(0.286)	(0.259)	(0.238)	(0.312)	(0.271)	(0.243)
ssa	−0.599***	0.248	0.323*	−0.682***	0.332*	0.396**
	(0.210)	(0.193)	(0.186)	(0.215)	(0.187)	(0.181)
gn	0.198	−0.131	−0.128	0.375	−0.377	−0.375
	(0.307)	(0.271)	(0.262)	(0.410)	(0.310)	(0.303)
siml	0.285***	−0.221***	−0.228***	0.323***	−0.236***	−0.248***
	(0.0801)	(0.0710)	(0.0682)	(0.0895)	(0.0748)	(0.0717)
iq_i	−0.479***	0.399***	0.407***	−0.521***	0.456***	0.457***
	(0.0495)	(0.0484)	(0.0471)	(0.0564)	(0.0541)	(0.0510)
iq_j	−0.122***	0.102***	0.101***	−0.172***	0.155***	0.153***
	(0.0414)	(0.0367)	(0.0355)	(0.0484)	(0.0392)	(0.0383)
inst_dis	−0.0859**	0.0477	0.0424	−0.0578	0.0169	0.0195
	(0.0378)	(0.0367)	(0.0360)	(0.0413)	(0.0370)	(0.0359)
sigma2_u	0.190**	0.132	0.115	0.163**	0.0702	0.0630
	(0.0845)	(0.0917)	(0.0877)	(0.0814)	(0.0724)	(0.0712)
logs		−0.538***	0.0141		−0.556***	−0.00769
		(0.0568)	(0.0510)		(0.0561)	(0.0507)
Constant	53.09***	−44.61**	−43.79**	4.583	−14.09	−8.702
	(20.36)	(17.33)	(17.03)	(42.13)	(34.98)	(35.01)
Observations	506	506	506	459	459	459
Number of id	186	186	186	164	164	164

Standard errors in parentheses, *** p < 0.01, ** p < 0.05, * p < 0.1; Columns (1)–(3) present the estimated coefficients (standard errors in parentheses) for the 2000–2024 sub-sample, while columns (4)– (6) present the corresponding estimates for the 1997–2019 sub-sample.

The comparison of Table 4 with Tables 5 and 6 highlights notable robustness features and some sensitivity to the destination sample. The consistency in both specifications suggests that greater bilateral similarity, as captured by SIMIL, is associated with lower trade survival when the destination pool includes a broad set of countries but remains similarly negative when restricted to Global South destinations. This pattern implies that the relation between similarity and persistence is not driven solely by a subset of destinations outside the Global South; instead, the presence of similar partner characteristics may coincide with conditions that reduce persistence across both samples. The GDP-population controls (${POP}_{i},\;{POP}_{j}$) and distance maintain their expected signs, reinforcing the role of market size and proximity, while Inst_Dis remains positive in both specifications albeit with reduced magnitude in Table 5, indicating that institutional distance may matter but its impact is sensitive to the destination sample.

The destination-specific results for institutional quality ($IQ_{i},IQ_{j}$) and the regional dummy for COMESA reinforce a coherent core: institutional strength on both sides continues to promote trade survival, and the COMESA coefficient becomes statistically significant when the comparison is restricted to SSA. This suggests that COMESA’s positive contribution emerges within SSA, where contextual differences are less blurred by broader regional heterogeneity. Although the reduced sample size (from N=539 to N=486) widens standard errors slightly, the qualitative direction of the key coefficients remains intact. The findings thus support the robustness of the main conclusions: stronger institutions matter for persistence, and the country-similarity effect—negative in our specifications—persists, and its magnitude appears unresponsive to sample composition.

## 5. Conclusion

This study analyzes the longevity and stability of Ethiopia’s bilateral trade partnerships by examining the factors that shape the duration of these relationships. Using comprehensive trade data, gravity-based variables, and institutional indicators, it covers nearly three decades (1997–2023) to illuminate what sustains durable trade connections and what risks disrupt them. The findings show substantial heterogeneity in trade longevity: many relationships terminate early, while a subset persists for long periods. This pattern underscores that while establishing initial links is important, maintaining them requires navigating dynamic factors that influence trade stability. Larger export flows are associated with longer-lasting ties, suggesting that deeper economic integration and stronger market commitments bolster durability.

Institutional quality on both sides emerges as a critical determinant of longevity. Stronger institutions reduce transaction risks, improve contract enforcement, and facilitate smoother logistics, thereby extending trade durations. Interestingly, greater institutional distance between Ethiopia and partner countries is linked to longer survival, implying that complementarities in institutional arrangements—not merely similarity—can strengthen durable partnerships. Geographic factors also matter: sharing a border with Ethiopia markedly increases the survival probability of trade ties, reflecting lower transport costs and better logistical connectivity, while greater geographic distance tends to shorten durations. Moreover, regionally, trade within Sub-Saharan Africa shows relatively higher longevity, likely due to improved information flow and regional integration efforts. Economic size and population effects are asymmetric: Ethiopia’s larger GDP correlate with shorter survival, whereas a higher GDP in partner countries is associated with longer durations.

Policy-wise, the study recommends strengthening institutional quality through governance reforms and trade facilitation to reduce transaction costs and build partner trust. Significant investments in transportation and digital infrastructure are essential to improve connectivity and alleviate logistical bottlenecks. Ethiopia should intensify engagement in regional trade agreements, such as COMESA and AfCFTA, to reap the benefits of market integration and regulatory harmonization.
